# How and why music therapy reduces distress and improves personhood for people with dementia, staff and families on NHS mental health dementia wards: a realist evaluation

**DOI:** 10.1186/s13690-026-01865-8

**Published:** 2026-03-03

**Authors:** Naomi Thompson, Helen Odell-Miller, Chris Pointon, Benjamin R Underwood, Emma Wolverson, Rachel Hunt, Abdulwarrith Olawale, Lucy Pickering, Alison Wilkinson, Christine Wise, Ming-Hung Hsu

**Affiliations:** 1 Cambridge Institute for Music Therapy Research, Anglia Ruskin University, Cambridge, UK; 2 Arts Therapies Services, Cambridgeshire and Peterborough NHS Foundation Trust, Fulbourn Hospital, Cambridge, UK; 3 Public Contributor, Cambridge Institute for Music Therapy Research, Anglia Ruskin University, Cambridge, UK; 4 Inpatient Dementia Experience Group, University of West London, London, UK; 5 Cambridgeshire and Peterborough NHS Foundation Trust, Fulbourn Hospital, Cambridge, UK; 6 Department of Psychiatry, University of Cambridge, Cambridge, UK; 7 Faculty of Science and Engineering, Anglia Ruskin University, Cambridge, UK; 8 Dementia UK, London, UK; 9 Geller Institute of Ageing and Memory, University of West London, London, UK; 10 East London NHS Foundation Trust, London, UK

**Keywords:** Realist evaluation, Programme theory, Music therapy, Mental health dementia care, Distress, Co-design

## Abstract

**Background:**

Mental health dementia wards care for people with dementia experiencing the most acute and complex distress but research into psychosocial interventions to manage symptoms is limited. Music therapy may be helpful, but access and practice vary. A novel music therapy intervention (MELODIC) was co-designed for these wards, informed by programme theory for music therapy with people with advanced dementia in institutional care. This theory should be refined for mental health dementia wards.

**Methods:**

A co-designed realist evaluation was conducted using mixed methods data from the MELODIC feasibility study with two National Health Service (NHS) mental health dementia wards. MELODIC was delivered on each ward for four weeks. Realist interviews were conducted post-intervention. Interventionist diaries and study documentation were recorded. Quantitative data, including patient, staff and family outcomes and routinely collected data, were gathered twice both pre- and post-intervention. Template analysis was conducted in NVivo to refine, test and adapt the initial programme theory. This was consolidated with previous research and stakeholder consultations.

**Results:**

The mid-range theory outlines the intervention components and contextual elements required for music therapy to trigger changes in patient, staff and family reasoning and behaviour which lead to observable outcomes. A qualified music therapist can assess and attune to individual unmet needs, supporting a short-term reduction in distress and improved sense of personhood. Additionally, the therapist works collaboratively with staff and families, with reciprocal communication, structures for knowledge exchange and support from management. This enables staff and families to use personalised music in their individual practice to support patient care and create a more therapeutic ward atmosphere, facilitating a positive change in ward culture and approach to distress management over time.

**Conclusions:**

This realist evaluation provides theory for how and why the MELODIC music therapy intervention can reduce distress and improve personhood for patients on NHS mental health dementia wards, with positive associated outcomes for staff and families. Further research should establish the efficacy and effectiveness of the intervention, with continued iterative refinement of the programme theory.

**Trial registration:**

ISRCTN86317609, registration date 25/04/2025.

**Supplementary Information:**

The online version contains supplementary material available at 10.1186/s13690-026-01865-8.

**Table:** 

Text box 1. Contributions to the literature
• Mental health dementia wards care for people with dementia experiencing extreme distress. Little is known about how to care for this clinical population.
• MELODIC is a co-designed music therapy intervention for these wards informed by evidence and programme theory. The theory was refined using mixed methods data.
• The mid-range theory outlines the music therapist’s role in assessing how music can be used to reduce distress and improve personhood. When embedded in the team, they support the use of personalised music in everyday care, facilitating positive changes in the ward culture.
• This theory should inform psychosocial intervention implementation in dementia care.

## Background

Mental health dementia wards care for people with dementia experiencing crisis, including extreme and multifaceted distress, which is putting their safety or the safety of others at risk [1, 2]. Distress refers to behavioural changes often experienced by people with dementia which can be caused by symptoms of dementia and/or be an expression of unmet need [3–6]. Presentations may include agitated behaviours such as shouting, throwing, hitting and kicking, or non-agitated behaviours such as pacing with purpose, crying, withdrawal, or resistance to care/medication [7]. Care provision varies internationally, but in England and Wales people with dementia may be detained on National Health Service (NHS) wards under appropriate legislation [8]. Admission often follows a breakdown in care and wards aim to assess and alleviate the crisis through reviewing medication, developing care plans, and providing suitable care packages post-discharge [4, 9–11]. Evidence-based recommendations state that psychosocial interventions should be the first line of treatment for distress behaviours [12]. However, there is limited research with varying methodological quality into psychosocial interventions on mental health dementia wards internationally [13]. Additionally, pharmacological interventions are frequently used despite evidence of limited benefit and common and severe adverse effects, including physical illness and death [13–18]. Psychosocial interventions, in particular music therapy and multisensory interventions, may reduce distress when delivered on mental health dementia wards by trained interventionists in a person-centred, accessible way [13]. However, barriers to implementation include the absence of trained interventionists, limited staff time and high levels of staff turnover [13]. 

Music therapy is a psychosocial intervention delivered by a registered, accredited therapist, recognised in best practice guidelines as a therapy that could support wellbeing for people with dementia [14]. Systematic reviews suggest that music therapy could reduce agitation, depression and distress behaviours for this clinical population, although there is a lack of high quality evidence [19, 20]. Research conducted on NHS mental health dementia wards suggests that, while few wards have access to music therapy, the intervention is feasible and could reduce the prevalence of distress behaviours [21–25]. However, due to a lack of time on the ward, music therapists often provide group sessions only, with limited individual assessments or engagement with multidisciplinary teams and families to support the use of music in everyday care [21–25]. While staff on NHS mental health dementia wards value music as an important part of care, its use is often ad hoc, not always personalised and staff report limited understanding of music therapy [26]. 

**Fig. 1:**
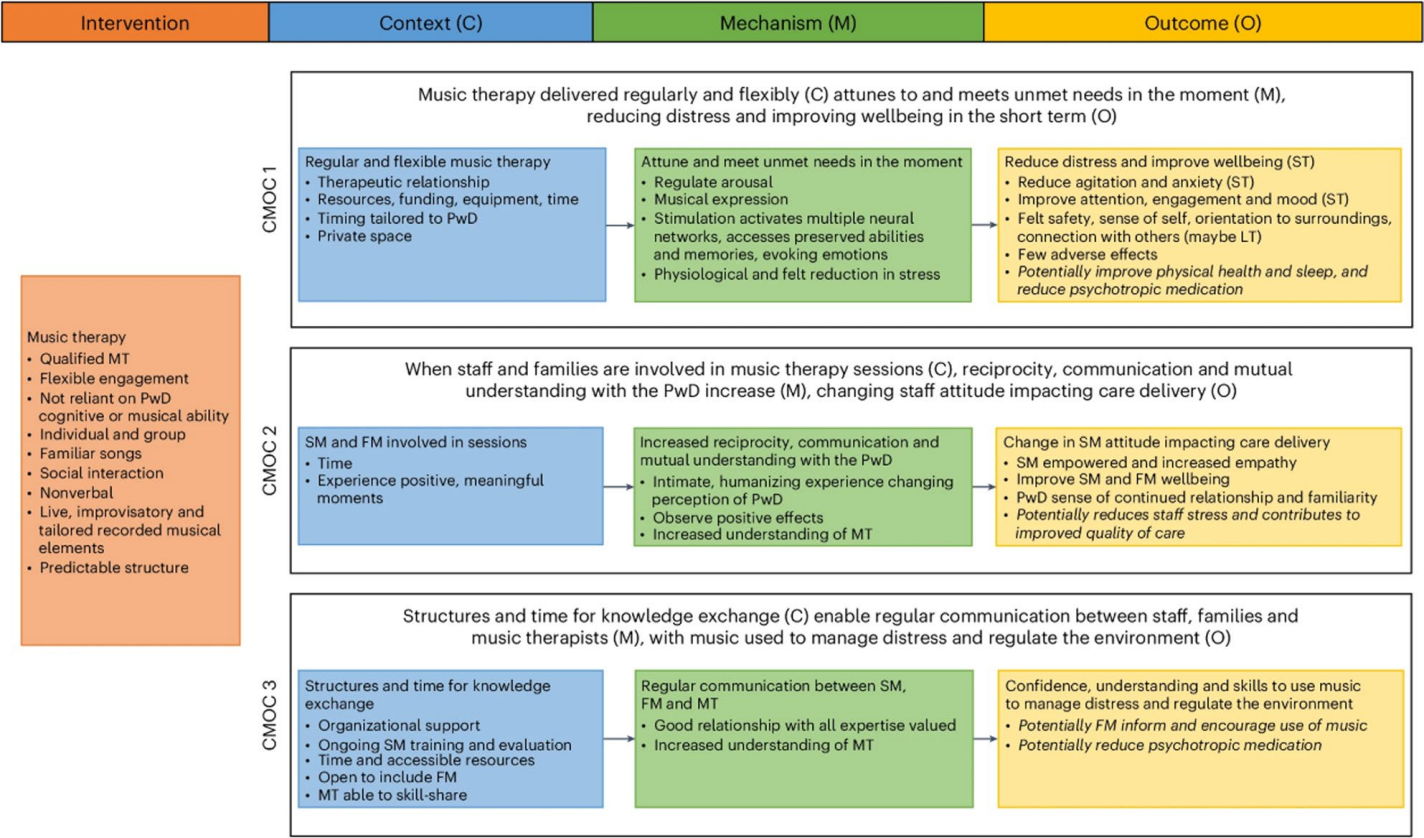
Programme theory for how and why music therapy reduces distress and improves wellbeing for people with advanced dementia in institutional settings, developed through a realist review. Reproduced with author permission Thompson et al., 2024 [30]. Statements in italics indicate where the supporting evidence is weak. MT = Music therapist; SM = Staff member; FM = Family member; PwD = Person with dementia; ST = Short term; LT = Long term

Realist research is theory-based, identifying how interventions interact with the wider context to cause observable outcomes [27]. The interaction between the intervention (also known as resource), and the context, triggers hidden mechanisms (i.e. changes in reasoning and response) which generate intended and unintended outcomes [28, 29]. A programme theory for an intervention aims to outline ‘what works, for whom, in what circumstances’ presented as context-mechanism-outcome configurations (CMOCs) [28]. Our team previously conducted a realist review, synthesising existing evidence to further understanding of the contextual factors and mechanisms underlying how and why music therapy may reduce distress and improve wellbeing in advanced dementia care [30]. The programme theory suggested that where music therapy was delivered regularly and flexibly, the music therapist could attune to and meet the person with dementia’s unmet needs, leading to short-term reductions in distress and improvements in wellbeing [30]. Additionally, when staff and families were involved in music therapy sessions, reciprocity and mutual understanding increased, thus shifting staff attitudes and influencing how care was delivered. Structures and time for knowledge exchange also enabled regular communication between staff, families and the music therapist leading to increased staff confidence to use music to manage distress and regulate the environment (Fig. 1). In contrast to a realist review, a realist evaluation investigates these mechanisms in practice. A realist evaluation is particularly suited to identifying how contextual factors shape mechanisms that produce outcomes in complex psychosocial interventions, which cannot be examined through an RCT design alone.

The programme theory developed through the realist review informed the co-design of a music therapy intervention protocol for mental health dementia wards called MELODIC (Music therapy Embedded in the Life Of Dementia Inpatient Care) [31]. The key components of the MELODIC intervention were: (1) a music therapist embedded in the multidisciplinary team (MDT) for 15 h a week; (2) delivery of specialist individual and group music therapy sessions; (3) development of musical care plans for each patient; and (4) support and training for staff and families to implement musical care plans (Supplementary File 1 for intervention protocol). Principles of practice for the music therapist were outlined including: collaborating with staff and families; flexible delivery of interventions; assessing for triggers of distress and unmet needs; and being aware of the potential to trigger a negative response. Three two-hour interactive, online training sessions for the interventionists were developed, with weekly one-hour supervision online during intervention delivery. The complex intervention development study, including a feasibility study on two wards, was funded by the National Institute for Health and Care Research (NIHR204928) [31, 32]. Here we report a realist evaluation using data gathered from the MELODIC feasibility study to refine the initial programme theory and develop mid-range theory tailored to the unique contextual factors present in NHS mental health dementia wards. In simple terms, a mid-range theory shows how and why the intervention works for this group in this context and is developed through empirical research to test and refine initial programme theory. All research activities comply with ethical regulations and were approved by the Health Research Authority (IRAS, no. 323503), and Anglia Ruskin University (ETH2223-8044). The following research questions were co-designed and adapted from the realist review:

1. What are the components and mechanisms of music therapy interventions that reduce distress behaviours and improve wellbeing for people with dementia on NHS mental health wards?
2. What are the components and mechanisms of music therapy interventions that improve the quality of care delivery and wellbeing for staff and family members?
3. What are the factors influencing successful implementation of music therapy interventions on NHS mental health dementia wards?

## Methods

A realist evaluation was conducted to refine and test programme theory for how and why music therapy reduces distress and improves wellbeing for people with advanced dementia in institutional settings, developed through a realist review. The theory was treated as an initial programme theory and tailored to NHS mental health dementia settings based on data from a feasibility study of the MELODIC intervention, creating a mid-range theory. The evaluation supported adherence to the Medical Research Council guidelines for developing complex interventions and is reported in line with RAMESES II reporting standards (Supplementary File 2) [33, 34]. This was a co-designed project with a research team of academics, clinicians and experts-by-experience working collaboratively on all stages of research design, data collection, analysis, interpretation and dissemination [35, 36]. The co-design group included a ward manager, a nurse consultant, an occupational therapist, a music therapist, a psychiatry trainee, and two family carers, who met as needed to review emerging findings and refine intervention components.

### Study design

**Fig. 2:**
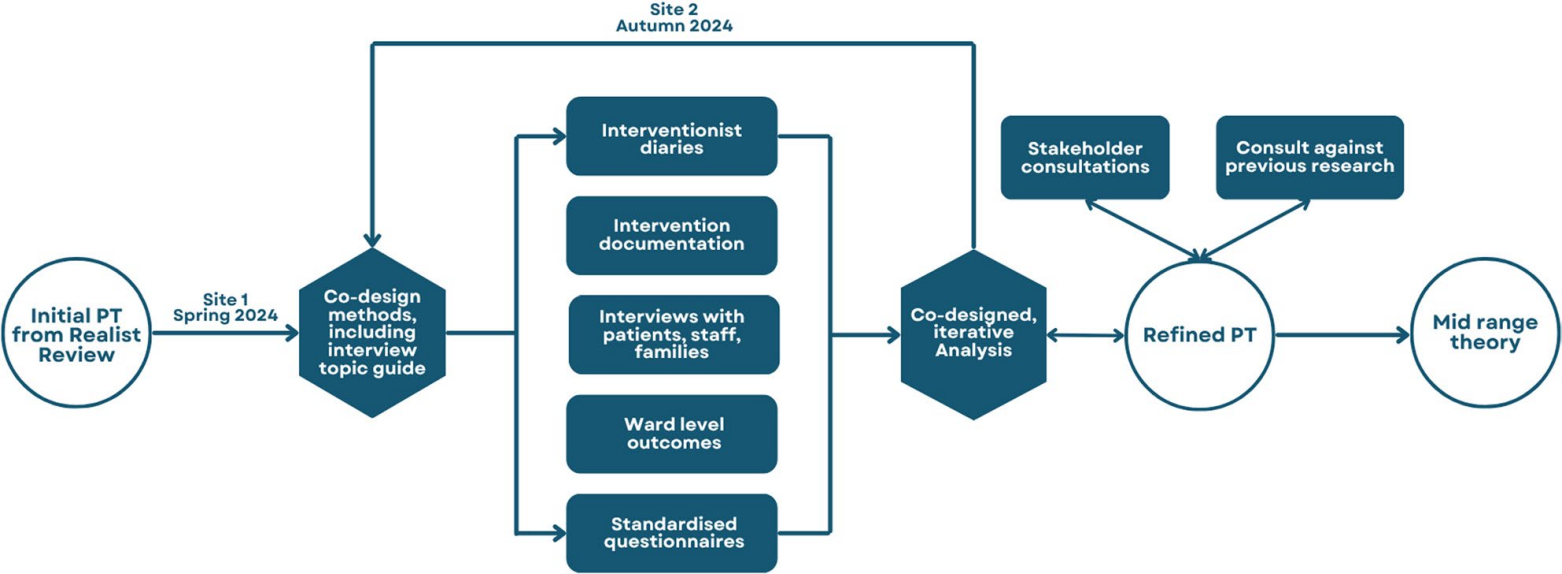
Methods for realist evaluation. Iterative, co-designed methods used to refine the initial programme theory. PT = Programme Theory

Mixed methods data were collected in a feasibility study of the MELODIC intervention on two mental health dementia wards consecutively in different NHS Trusts in England (Fig. 2). Different music therapists delivered the intervention at each site for four weeks. The four-week period balanced the need for sufficient intervention exposure with the practical time constraints of an acute mental health dementia ward, taking into consideration the average patient length of stay. Wards were recruited purposively to test intervention feasibility and acceptability in settings with differing experiences of music therapy. MELODIC version v1 was piloted on site 1, a 14-bed inpatient unit which was already providing a weekly music therapy group, in Spring 2024. Qualitative data were used to refine the intervention protocol alongside the co-design group. MELODIC v2 was piloted on site 2, a 16-bed inpatient unit which had never delivered music therapy, in Autumn 2024. Data were again used to support intervention refinement with the co-design group, creating MELODIC v3.

### Data collection

Qualitative data were gathered through realist interviews with patients, families and staff from both wards in the two weeks after the pilot [37]. Interviews were conducted individually by NT and MHH in-person on the ward or on Microsoft Teams, and audio recorded and transcribed by NT. As interviews were conducted by music therapists, reflexive memoing and discussion within the co-design team were used to enhance transparency and reduce potential bias. Semi-structured interviews were used, tailoring the content to the experience and expertise of the participant. The topic guide was co-designed to refine the initial programme theories, including outcomes, resource requirements, mechanisms, and contextual factors. Elements of the CMOCs were presented for comment in line with teacher-learner roles [37]. A practice interview was conducted prior to data collection with a member of the co-design team to ensure accessibility of the language used and helpfulness of the questions. As the two pilots occurred consecutively, the topic guide was adapted with the co-design group after initial analysis of data from site 1 to test emergent theories (Supplementary File 3).

Additionally, the music therapists’ diaries, outlining the time, length and description of all interactions with patients, staff and families, were analysed, with qualitative and quantitative data extracted. Staff feedback forms following training (site 1 only) and after music therapy sessions (site 2 only) were also analysed, alongside patient musical care plans and email communications between the music therapist and the staff team (Supplementary File 1 for blank forms). Finally, quantitative data from the ward level outcomes and standardised questionnaires were included to further refine the programme context and outcomes for patients, staff, families, and the ward overall. Standardised questionnaires looking at patient, family and staff outcomes were collected at four timepoints (1 month historical, baseline, endpoint and 1 month follow up) with ward level data collected at the end of the study period (see protocol ISRCTN86317609 and feasibility paper for description of collected data) [31, 32]. 

Purposive sampling was employed to include people with varying roles and engagement with the intervention including patients, families, managers, clinical staff, therapy staff, doctors and administrative staff. All participants who had consented to take part in the feasibility study were eligible regardless of the number of music therapy sessions attended. This included patients for whom consultee agreement had been provided by next-of-kin or responsible clinician. In addition, participants could provide consent to take part in the interviews only.

### Data analysis and theory consolidation

All data were inputted to NVivo for analysis, with memos used to track decision making and improve transparency in discussions with the co-design team [38, 39]. Data from site 1 were used to refine the initial programme theory, which then guided the deductive analysis of data from site 2. This sequencing was designed to clarify the purpose of each data type and ensure a coherent synthesis across the two sites. In line with realist evaluations, analysis occurred iteratively as data were collected, influencing subsequent data collection [40]. Realist interviews were analysed drawing on the principles of template analysis, a type of thematic analysis using a combination of inductive and deductive coding and development of themes, in keeping with the realist method of retroduction [27, 41]. Following familiarisation with data from site 1, transcripts were coded inductively by NT to create a coding template. Codes were then grouped into themes based on the initial programme theory which formed a priori themes, with all proposed changes documented in a live memo for each CMOC. This was reviewed and discussed in depth with MHH and EW, with changes subsequently made to the interview topic guides based on the proposed revisions to the programme theory. Qualitative data from site 2 were then coded deductively using the coding template, with any additions, refinements or disagreements in the data recorded in the related memo for each CMOC. Changes were again discussed with EW and MHH. Once all quantitative data were collected, these were inputted into NVivo and coded to support, refute or refine relevant aspects of the CMOCs, particularly in relation to context and outcomes. Theory changes that were not consolidated by multiple sources, for example in both qualitative and quantitative data, were marked as having weak supporting evidence.

The proposed, refined programme theory was shared with the co-design group for refinement and consolidation. Additionally, the authors reviewed the theory considering findings from a qualitative study conducted during a previous work package in the MELODIC study including 49 patients, families and staff on NHS mental health dementia wards [26]. The qualitative study explored experiences and management of distress and ways music and music therapy was or was not used. Once consensus was achieved, the theory was presented to participants from the two pilot sites alongside patient, public and professional consultations to sense-check and identify gaps. All feedback were recorded and incorporated in italics into the theory for sharing with further groups and recorded on the memos in NVivo. Changes were related to clarification of wording and content alongside evidence supporting the programme theory. All changes were consolidated with the co-design group to produce the mid-range theory.

## Results

Across both sites, 28 patients were recruited alongside 13 family members and 48 staff members (for patient baseline characteristics see Table 1; for staff and family member characteristics see Supplementary File 4). Of these, 42 participants took part in interviews following the intervention, including five patients, five family members and 32 staff members. Four staff members and one family member consented only to interview and not the questionnaires. Questionnaires exploring patient, staff and family outcomes were collected with 94.3% data completeness across four timepoints. Please refer to the feasibility results paper for a full description of the quantitative data [32]. 

**Table 1:** Patient baseline characteristics *n* = 28. Demographics for patients who took part in the interviews are not separated due to small numbers to protect patient anonymity

Age	
Mean	76.88
Range	26.00
No. female	15
No. male	13
Religion	
Christian	16
Not stated	8
None or other	4
Ethnicity	
White British	28
Diagnosis	
Alzheimer’s	8
Mixed dementia	7
Ongoing assessment	6
Vascular dementia	3
Unspecified dementia	2
Other specific dementia diagnosis	2
Time spent on the ward (weeks, mean)	10.02
Time since diagnosis (years, mean)	2.86
Admitted from	
Own home	9
Full time residential care	7
General hospital	10
Not stated	2
Other co-existing mental health diagnoses (mean no. per patient)	0.45

### Mid-range programme theory

**Fig. 3:**
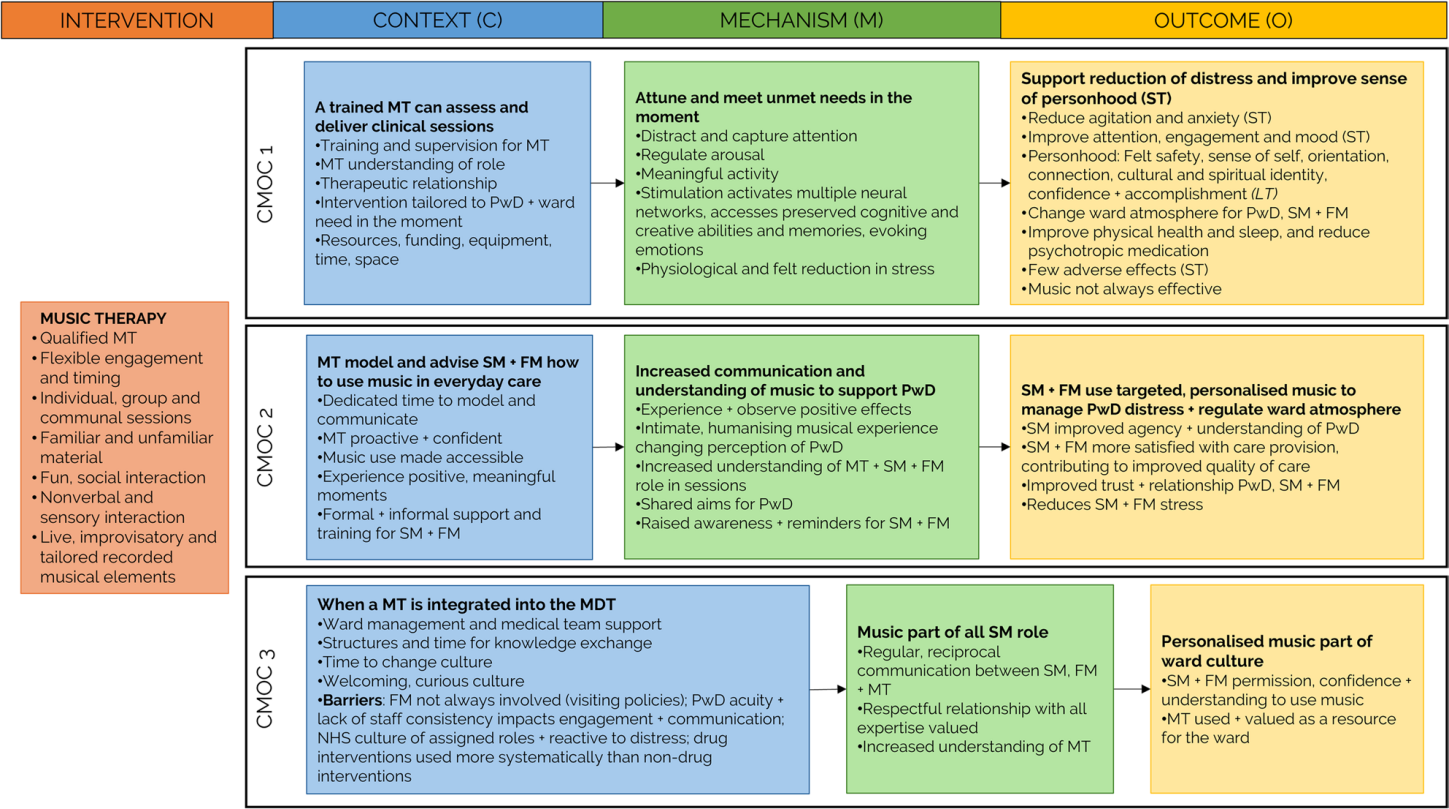
Mid-range programme theory for the MELODIC music therapy intervention for NHS mental health dementia wards. C = Context; M = Mechanism; O = Outcome; MT = Music therapist; PwD = Person with dementia; SM = Staff member; FM = Family member; MDT = Multidisciplinary team; ST = Short-term; LT = Long-term

The following mid-range programme theory was developed and refined with the co-design team using qualitative and quantitative data from the feasibility study, multiple key stakeholder consultations, and previous qualitative research (Fig. 3, supporting qualitative and quantitative data are provided in Supplementary File 5).

#### Intervention: music therapy

Music therapy is delivered by a qualified and registered therapist. The therapist delivers individual and group clinical sessions including musical, sensory and verbal interactions such as singing songs, music listening, improvisation and instrumental play, dancing and cognitive stimulation. They also advise and support the use of staff-led music activities, such as singing, listening to recorded music and instrumental play, in communal areas of the ward. Musical interactions are accessible regardless of cognitive and physical impairments, with patients able to engage in verbal and nonverbal interactions, using a variety of live and recorded musical elements in active and passive ways. Sensory interactions, such as materials stimulating tactile and olfactory senses and interventions supporting movement, are also important for this clinical population who are often in the advanced stages of dementia. Music can be tailored for different purposes to create a fun and lively atmosphere or to have a calm, relaxed space. Familiar music can create a sense of safety, while unfamiliar music can spark interest and shared experiences. Musical tastes may change as the person’s dementia progresses.

#### CMOC1: A trained music therapist can assess and deliver clinical sessions (C) which attune to and meet unmet needs in the moment (M) supporting a reduction in distress and improving sense of personhood (O)

All patients should receive a music therapy assessment focussing on identifying causes for distress and unmet biopsychosocial needs, with ongoing clinical individual and/or group sessions provided as required. The therapist should prioritise those presenting with the most complex distress and/or during the early stages of admission. A therapeutic relationship, including knowledge of musical tastes, is required for the patient to engage in therapy and the therapist to tailor the intervention to their needs in the moment. Consideration of the timing and length of clinical sessions to maximise effectiveness and avoid over-stimulation is needed. Assessments made through observing responses and engagement with musical and sensory stimuli provide a different perspective on the patient’s cognitive, psychological and physical needs, contributing to the MDT’s understanding. This requires collaboration and communication with staff and families. The therapist can also assess for ways music could support the whole ward including advising on the appropriate use of recorded music in communal areas. The music therapist needs training and ongoing supervision in the MELODIC intervention and working within a multidisciplinary approach to support treatment adherence and problem solving. The music therapist should be employed on a permanent contract, with adequate resources, equipment and time. Different spaces on the ward can be used, with sessions in private rooms enabling in-depth psychological work while sessions in communal areas impact the ward atmosphere and provide potential for spontaneous interactions.

The music therapist can meet unmet needs in the moment by distracting and capturing attention, regulating arousal, providing meaningful activity and stimulation, and reducing stress. Distraction, using familiar songs, instrumental music and rhythm, is often the first stage of arousal regulation, reducing stress and early signs of distress. Providing interventions that are meaningful, engaging and stimulating help to lift mood and support personhood, preventing distress. The neural mechanisms stimulated through musical interactions enable access to preserved cognitive and creative abilities and salient memories, evoking emotions and supporting verbal and nonverbal expression.

Music therapy supports the prevention and reduction of distress, including agitation and anxiety. The length of the impact varies from relief in the moment to changes in presentation for the rest of the day depending on the individual and external factors on the ward. When the patient and ward are regulated and calm, there is increased focus, sustained attention and engagement which can lift mood. Music therapy also supports the individual’s sense of personhood recognising their value and individual identity. This was expressed through a felt sense of safety, sense of self and their cultural and spiritual identity, orientation to surroundings, connection with patients, families and staff, and feelings of confidence and accomplishment. This increased sense of personhood can be long-term and is unique to music therapy and music interactions. Music therapy can also change the ward atmosphere, with the ability to create times of calmness as well as lively interactions. These outcomes could lead to improvements in patients’ health, with increased compliance with care interventions, improved sleep quality for some, and potentially a reduction in the use of prescribed psychotropic medication. Additionally, while there may be negative responses to music in the moment, including triggering upsetting memories or overstimulation, the music can be stopped and support provided with no lasting adverse impact. However, staff note that music is not always effective with restrictive interventions needed when the patient is in a high state of distress.

#### CMOC2: Music therapists’ model and advise staff and families how to use music in everyday care (C) increasing communication and understanding of music to support people with dementia (M) with targeted, personalised music used to manage distress and regulate the ward atmosphere (O)

Following assessment, the music therapist should demonstrate to staff and families ways to incorporate music into care activities through modelling interventions and formal and informal communication. This requires the therapist to be proactive and confident, and to have dedicated time to document assessments, attend team meetings, write care plans and spend time with staff and families. Support and training for staff and families should build on ways music is already being used and considering what is feasible for staff to implement. Additionally, music use should be made accessible, with equipment available and an understanding of what music to use when for each patient. This was supported by staff and families observing and joining in music therapy sessions and experiencing positive, meaningful moments with patients. The amount of time and support staff and families need will vary depending on their familiarity with music therapy and the acuity of the patients. The type of support will also depend on the ward and the role of the staff member. While informal workshops may be more appropriate and feasible for ward-based staff to engage with, ward management require an understanding of the intervention and its purpose within the overall ward aims. Support for families should be individualised, with additional support provided on admission and when attending clinical sessions.

Interactions with the music therapist support increased communication and understanding of ways music can be used to support patients in a setting where staff are highly skilled and value the importance of personalising care. Observing the positive impact of music therapy and colleagues employing musical interventions encourage and motivate staff to try interventions themselves. Musical experiences are often intimate and humanising, helping to change perceptions and see a different side of the patient. Reciprocal communication supports shared aims for the patient, with the music therapist becoming aware of where support is most needed and staff and families understanding the purpose for the music interventions. This raises awareness and provides reminders to use music to support patient care.

Where these mechanisms are activated, staff and families use targeted, personalised music to help manage patient distress and regulate the ward atmosphere. This is particularly evident during one-to-one observations, personal care interventions such as washing, dressing, mealtimes and night times, and to have meaningful engagements. Staff and families have an improved sense of agency, with an increased understanding of the patient, potential triggers for distress and ways to support them when they are unsettled. There are also improved relationships and trust between the patients, families and staff and enhanced satisfaction and felt quality of the care delivered. Overall, staff are less stressed with care tasks being easier and more interactive to deliver. Families reported being less anxious when they feel their relative is receiving more personalised care.

#### CMOC3: When a music therapist is integrated into the multidisciplinary team (C) music becomes part of all staff members’ roles (M) enabling personalised music to become part of the ward culture (O)

The music therapist should be integrated into the MDT to tailor and embed music interventions within the resources and capabilities of the ward at the time. Music therapy assessments should be informed by and, in turn, inform the wider MDT’s assessments of the individual’s needs, development of care plans, and plans for discharge alongside families. Integration requires support from the ward manager and consultant psychiatrist to facilitate communication and support staff to incorporate musical interactions in their care. Ward management are also responsible for creating a welcoming and curious culture where staff feel able to try new things and share ideas. Structures and time for knowledge exchange are crucial. Structures include formal and informal meeting spaces, named link people to facilitate communications, integration of clinical notes detailing music therapy interventions and outcomes into the patient record, and clear, visible and consistent documentation in musical care plans. Time is required for the novel music therapy intervention to become familiar and embedded in practice.

However, there are barriers to integration. Family members are not consistently involved in their relatives’ care and are constrained by the ward’s visiting policies. The fluctuating number and acuity of patients on the ward impacts staff ability to engage and communicate with the therapist and think about new ways of providing care, at times contributing to feelings of hopelessness. This is exacerbated by a lack of consistency in staffing with frequent use of agency staff. In addition, there is a prevailing culture of staff having assigned roles in the NHS preventing them from feeling responsible or able to explore alternative, psychosocial ways to support distress symptoms. Finally, there can be a biomedical and reactive approach to distress, with drug interventions implemented more systematically than psychosocial interventions and a resistance to implementing preventative interventions.

When music therapy is embedded, music could become part of all staff members’ roles and responsibilities. This is supported by regular, reciprocal communication between the music therapist, staff and families to continually refine how music is used to reduce distress and improve personhood as a patient’s needs change throughout the admission. A respectful relationship between all parties develops, with the different expertise of all valued alongside a shared understanding of the role and purpose of music therapy in the MDT.

Where music is part of everyone’s role, personalised music becomes part of the ward culture. The role of families in informing and encouraging the use of music for their relative is respected, while staff are the main actors in incorporating musical interactions in everyday care interventions. Staff and families have the confidence, understanding and permission to use music in responsive and interactive ways depending on individual and ward need, as well as an awareness of the potential for overstimulation. The role of the music therapist is valued by families and the MDT. This includes delivering specialist assessments of patient need and causes for distress, incorporating music into patients’ everyday care, providing interventions to reduce distress and support personhood in the moment, and contributing to discharge plans. Overall, this supports the ward aims and takes pressure off staff in a complex and challenging care environment.

## Discussion

This realist evaluation refines previous programme theory for music therapy in institutional dementia care for people with advanced disease, tailoring it to NHS mental health dementia wards to produce a mid-range programme theory. It provides a clear and detailed description of how qualified music therapists, trained in MELODIC principles, can work with patients, staff and families to embed the personalised use of music in everyday care to support distress management, including the contextual factors required. This provides a theory-based approach to the MELODIC music therapy intervention in a setting where practice is limited and varied, supporting implementation in a complex healthcare system [25]. 

The mid-range theory was developed through a rigorous co-design process including qualitative and quantitative data based on realist methodology [28]. The employment of realist interviews with iterative data analysis enabled gaps in the initial programme theory to be filled, and emergent theories to be tested and consolidated [30]. Inclusion of quantitative data exploring ward and individual outcomes assisted theory refinement. Checking against previous qualitative research and multiple participant, patient, public and stakeholder consultations supported the clarity and refinement of the theory and highlighted areas of importance [26]. 

Mental health dementia wards provide care for the most unwell and distressed people with dementia for whom community care is not currently deemed appropriate [2, 14]. Therefore, the potential for music therapy to support the reduction of distress and positively change the ward atmosphere is important where evidence for psychosocial interventions is limited and restrictive interventions, such as psychotropic medication, are frequently used [2, 13]. Reductions in distress were accompanied by a greater sense of self, safety, and orientation in a setting where patients report a perceived lack of safety [26]. This can be described as an improved sense of personhood, aligning with Kitwood’s theory of person-centred care which states that, while cognitive and physical abilities will decline as dementia progresses, it is possible to maintain and uphold the individual’s sense of being loved and valued [42, 43]. These outcomes have potential health and cost implications with compliance with care interventions, improved sleep and reduced medication reported for some patients and supported by trends in the ward data. Staff stated that there were few and short-lasting adverse effects associated with music therapy which was shown in the ward data with no increase in routinely reported distress or safety incidents during the intervention period. This contrasts with common, severe and long-term adverse effects associated with psychotropic medication [16–18]. As the only mid-range programme theory for music therapy interventions, elements may be applicable to other settings and clinical populations. However, this would need to be tested and refined to ensure the theory remains relevant to the specific context and population.

Outcomes for patients were accompanied by positive outcomes for staff and families. Staff reported improved sense of agency, with additional tools to manage distress and engage with patients, and improved knowledge and relationship with the patient. The contextual requirements for knowledge exchange in CMOC2 and CMOC3 support previous research outlining that effective staff training combines classroom and practice-based teaching with ongoing supervision, provides relevant, structured tools, and has support from the organisation for attendance and implementation of new skills [44]. This has implications for delivery of care with research reporting that staff being able to personalise care to the patients’ needs was the most important way to help manage their distress and support readiness for discharge [26]. However, this was not reflected in the quantitative data potentially due to the short length of the intervention, the data collection tools, or the complexity of the continued pressures on the ward. Future research may benefit from measuring changes in staff competency and self-efficacy which have shown responsiveness to staff training interventions in dementia care [44, 45]. Additionally, both the quantitative and qualitative findings suggest that there was reduced stress and improved satisfaction for families. This is important given research highlighting the high levels of trauma and stress surrounding inpatient admission for families [46]. 

Our findings also sit within a broader evidence base examining the contextual conditions needed for music-based interventions in dementia care. While some recent studies of music interventions have highlighted barriers to session delivery, such as staffing constraints and organisational readiness, these largely focus on what happens within structured sessions and provide limited insight into how musical approaches become embedded in everyday care [47]. Rasing et al. discuss wider organisational factors, though their emphasis similarly centres on enabling the delivery of structured music therapy sessions [48]. In contrast, MELODIC aims to support a cultural shift in ward practice by building staff knowledge, confidence and shared understanding so that music can be used flexibly and purposefully throughout the day. The programme theory also reflects an expanded role for the music therapist, who works collaboratively with the ward team to identify root causes of distress, support problem-solving and strengthen communication pathways. This differs from traditional models focused on delivering sessions and highlights the importance of MELODIC training that equips music therapists with the skills needed to investigate challenges and co-develop solutions with staff and families. This aligns more closely with evidence from our systematic review of psychosocial interventions on inpatient mental health dementia wards, which emphasises the importance of staff capability, team communication and supportive organisational contexts for sustainable practice change [13]. 

The presented mid-range theory has similarities to the initial programme theory [30]. Data supported the core components of the music therapy intervention, the mechanisms through which music therapists can support distress reduction, and ways they can skill share with staff and families. However, there were also additions and changes to the initial programme theory. Some additions filled gaps identified in the literature into the impact of the infrastructural context on the delivery and outcomes of the intervention, outlined in CMOC3. Many changes also reflect the difference between mental health dementia care settings and other institutional care settings such as residential care. The shift from wellbeing to personhood reflects the complex presentation of patients, including palliative care needs, and the importance of maintaining a sense of the individual at a time when they are very unwell [1, 26]. These wards also have a higher staff: patient ratio than most care settings, led by skilled mental health nurses supported by the MDT. There is a focus on assessment, development of care plans and supporting timely discharge [9, 10, 26]. These contextual factors are reflected in the music therapist’s role being embedded in the MDT, contributing to assessments, and respecting and building on the team’s and families’ expertise. Music therapists will require training in the principles of MELODIC, ongoing supervision and understanding of this collaborative way of working to effectively deliver this music therapy intervention.

Barriers to implementation reflect the complexity of introducing a new intervention to a complex healthcare setting, supporting previously identified barriers to delivering psychosocial interventions in these settings [13, 26, 32]. Inconsistent staffing and fluctuating patient acuity highlight the need for the music therapist to be proactive and confident to tailor the intervention to the needs of the individual and the ward. Additionally, having clear structures to support communication, such as simple musical care plans placed in visible spaces, supported the transfer of knowledge within the ward team [44]. A culture of assigned roles, more structured approach to the use of medical than psychosocial interventions and focus on reducing rather than preventing distress was observed. To challenge this, support and understanding from the ward manager and medical consultant was needed for staff to feel required and permitted to utilise non-drug interventions, including music, in their practice, supporting research into psychosocial interventions in dementia care [49]. Our findings also support research highlighting the disparity in family involvement in mental health dementia care, which was largely attributed in our data to the ward visiting policy [11, 46]. This was reflected as fewer families chose to participate in the study despite being willing for their relative to participate. This supports the need for more research to provide guidance on how families can safely and helpfully be involved in care delivery [50]. 

## Limitations

There is potential for researcher bias as data were collected and initial analysis conducted by music therapists (NT and MHH). Mitigation was sought through recording all decision-making in memos, discussing the analysis in detail with the co-design group, and corroborating findings with literature and participant, patient, public and stakeholder groups. There is a lack of diversity in the patient and family participants and so the applicability of the findings to people accessing wards from diverse ethnic groups cannot be assumed. Further research implementing MELODIC on wards with a more diverse patient group is required. The theory focusses on mental health dementia wards within the NHS in the UK. While there may be similarities to mental health dementia care internationally and other dementia care settings, further research to refine and adapt to these settings is required. Additionally, the quantitative data available were not statistically powered to demonstrate the efficacy or effectiveness of MELODIC. Outcomes need to be tested through controlled trials to confirm or refute the trends shown.

## Conclusion

This realist evaluation refines initial programme theory creating a mid-range theory for a novel music therapy intervention, MELODIC, on NHS mental health dementia wards. The theory outlines the contextual factors required for the MELODIC intervention to trigger hidden mechanisms and so support reduction of distress, improve personhood and embed the use of personalised music in everyday care. This theory should inform practice and policy in NHS mental health dementia care with implications for psychosocial interventions in these settings. It should continue to be tested and refined through trials to measure the efficacy and effectiveness of MELODIC to reduce distress experienced by people with dementia on NHS mental health dementia wards.

## Supplementary Information

Supplementary Material 1.

Supplementary Material 2.

Supplementary Material 3.

Supplementary Material 4.

Supplementary Material 5.

## Data Availability

The datasets generated and/or analysed during the current study are not publicly available to due to their confidential nature but are available from the corresponding author on reasonable request.

## Abbreviations

CMOC: Context Mechanism Outcome Configuration
EW: Emma Wolverson
MDT: Multidisciplinary Team
MELODIC: Music therapy Embedded in the Life Of Dementia Inpatient Care
MHH: Ming–Hung Hsu
NHS: National Health Service
NT: Naomi Thompson
V: Version
