# Supplementary material for: How and why music therapy reduces distress and improves personhood for people with dementia, staff and families on NHS mental health dementia wards: a realist evaluation
Source: Arch Public Health. 2026 Mar 3;84:69. doi: 10.1186/s13690-026-01865-8 (PMC13064223; doi:10.1186/s13690-026-01865-8)
Supplement: Supplementary file 4 — Supplementary Material 4. [file 13690_2026_1865_MOESM4_ESM.docx]

## Staff and family baseline characteristics

Table 1 Staff baseline characteristics

| Job title |  |
| --- | --- |
| Healthcare Assistant | 15 |
| Nurse | 12 |
| Nursing Associate | 5 |
| Deputy Manager/Team Lead | 4 |
| Student Nurse | 2 |
| Assistant occupational therapist | 2 |
| Music Therapist | 2 |
| Ward Manager | 1 |
| Doctor | 1 |
| Ward clerk | 1 |
| Activity Worker | 1 |
| Medicines optimisation technician | 1 |
| Assistant psychologist | 1 |
| Age |  |
| Mean | 40.19 |
| Range | 42.00 |
| Gender |  |
| Female | 37 |
| Male | 10 |
| Other | 1 |
| Religion |  |
| Christian | 20 |
| Not stated | 10 |
| None | 7 |
| Catholic | 6 |
| Atheist | 2 |
| Sikh | 1 |
| Buddhist | 1 |
| Ethnicity |  |
| White British | 25 |
| Asian | 8 |
| Black African | 10 |
| Mixed or multiple ethnic groups | 3 |
| White European | 2 |
| No. working hours per week (mean) | 36.41 |
| Time working on the ward (months, mean) | 57.46 |
| Highest level of education**** |  |
| Level 3 | 6 |
| Level 4 | 0 |
| Level 5 | 3 |
| Level 6 | 20 |
| Level 7 | 12 |
| Not stated | 7 |

Table 2 Family baseline characteristics

| Age |  |
| --- | --- |
| Mean | 66.70 |
| Range | 20.50 |
| No. female | 8 |
| No. male | 5 |
| Religion |  |
| Christian | 7 |
| None | 3 |
| Not stated | 3 |
| Ethnicity |  |
| White British | 13 |
| Working status |  |
| Retired | 8 |
| Employed | 3 |
| Other | 2 |
| Relationship to patient |  |
| Spouse/Partner | 7 |
| Child | 3 |
| Other relation | 3 |
| Highest level of education |  |
| Level 3 - 4 | 5 |
| Level 6 | 4 |
| Level 7 | 3 |
| Not stated | 1 |
