# Supplementary material for: How and why music therapy reduces distress and improves personhood for people with dementia, staff and families on NHS mental health dementia wards: a realist evaluation
Source: Arch Public Health. 2026 Mar 3;84:69. doi: 10.1186/s13690-026-01865-8 (PMC13064223; doi:10.1186/s13690-026-01865-8)
Supplement: Supplementary file 5 — Supplementary Material 5. [file 13690_2026_1865_MOESM5_ESM.docx]

**Table 1 Supporting evidence for the mid-range theory.** Quotes from realist interviews, interventionist diaries and quantitative data from the feasibility study. No quantitative data reached statistical significance. Some data support multiple elements of the theory so may be presented more than once. Quantitative data and data from interventionist diaries are presented in italics for ease of identification. P1XXX indicates that participant was from site 1, P2XXX indicates that participant was from site 2. PwD = person with dementia, FM = family member, SM = staff member, MT = music therapist, CMAI = Cohen-Mansfield Agitation Inventory, NPI = Neuropsychiatric Inventory, QoL-AD = Quality of Life in Alzheimer’s Disease, ADQ = Approaches to Dementia Questionnaire, GHQ = General Health Questionnaire, IJS = Index of Job Satisfaction, MBI = Maslach Burnout Inventory.(1–7)

| **MUSIC THERAPY (Intervention)** |
| --- |
| “it's very helpful to have music therapist on the ward…Great to support patients and staff.” (P1001, SM)  “[the patient] doesn't vocalise much but he vocalises in music. It was really nice to see.” (P20017, SM)  “I think having more group sessions is quite helpful” (P1037, SM)  “it's proper one to one time you know rather than, sometimes with us we get called away or you're like busy sometimes it's difficult to properly sit” (P1007, SM)  “If it's familiar … That calm them down, that will help them.” (P1004, SM)  “Well, what I liked about it was it was fun and you were making people happy.” (P20028, PwD)   - *56% of music therapy sessions were unplanned and session time ranged across the working day (09:00 – 16:00)* - *The MTs recorded 18% of sessions as group sessions and 82% of sessions as individual sessions* - *Interventionist diaries recorded a wide range of musical, sensory and cognitive activities as part of clinical sessions* |
| **A TRAINED MUSIC THERAPIST CAN ASSESS AND DELIVER CLINICAL SESSIONS (CMOC1, context)** |
| “I think [the music therapist] is working well… I thought there was a good connection there between the two.” (P1032, FM)  “So, yeah, I've seen her, you know, being able to adapt it to the needs of the patient that she's seeing.” (P20020, SM)  “it is that dedicated time that [the music therapist] had what made the difference I feel.” (P20043, SM)   - *Both MTs attended 6-hours of online training prior to intervention delivery and one-hour weekly online supervision during intervention delivery* - *Both sites received the full allocated 15-hours per week of music therapy over the 4-week pilot* - *56% of music therapy sessions were unplanned and session time ranged across the working day (09:00 – 16:00). The length of sessions varied between sessions and between sites.* - *The MTs recorded 18% of sessions as group sessions, many of which were ad hoc, and 82% of sessions as individual sessions* - *Interventionist diaries reported clinical sessions taking place in a variety of ward spaces including different communal spaces and patient bedrooms* |
| **ATTUNE AND MEET UNMET NEEDS IN THE MOMENT (CMOC1, mechanism)** |
| “So when she's kind of losing her way a bit … but she can't really tell us what she wants, it just seems to help bring her back and focus her on something, and then she can articulate herself a bit better” (P20041, SM)  “it does seem to occupy them in, in a way that they enjoy” (P1024, SM)  “Music, you know, that that reaches the part … at the very back of the brain. So that part of the brain is still working, functioning for a very long time. And it reaches him.” (P1042, FM) |
| **SUPPORT REDUCTION OF DISTRESS AND IMPROVE SENSE OF PERSONHOOD (CMOC1, outcome)** |
| “even some of the most challenging patients … I've seen [them] even just sit and be calm for 10 or 15 minutes or so” (P1024, SM)  “Oh [I feel] happier inside, because I know I've shared something … And they'll remember it when they leave then, they'll have, you know, known what was going on” (P1028, PwD)  “Cause we've got a lot of poorly patients that are quite unsettled, it kind of brought them together for that period as well” (P20034, SM)  “But I do think group sessions … I feel like that's just time for everyone, even staff it just sort of, now breathe.” (P1037, SM)  “I've just come off nights. So using it with [a patient] to get him to settle, really good” (P1007, SM)  “I haven't seen any of them be agitated during a session” (P1024, SM)  “But there's always gonna be times where people are distressed and nothing's going to distract them from that, unfortunately. It's just the nature of this place isn't it.” (P20043, SM)   - *Trends in patient outcome data: mean agitation scores (CMAI) showing frequency of distress behaviours increased post-intervention, while number of patients displaying distress behaviours decreased post-intervention not maintained at follow-up. Severity of neuropsychiatric symptoms (NPI) decreased post-intervention, not maintained at follow-up. Quality of life (QoL-AD) improved post-intervention, not maintained at follow-up.* - *Recognition of personhood (ADQ) did not change for SM and increased for FM with the change maintained at follow-up* - *There were trends in a reduction in prescribed psychotropic medication use, but not PRN use, during the month of the MELODIC intervention at both sites* - *There was a trend in reduced numbers of staff-reported incidents of distress behaviours post-intervention, maintained at follow-up* - *There were no incidents of restraint or seclusion at site 2 during the intervention period – site 1 did not use restraint or seclusion* - *There was no increase in routinely reported incidents during the intervention period, and no adverse events were reported during the music therapy interactions or any staff-led music intervention* |
| **THE MUSIC THERAPIST MODELS AND ADVISES STAFF AND FAMILIES HOW TO USE MUSIC IN EVERYDAY CARE (CMOC2, context)** |
| “So she was kind of encouraging staff and showing us examples how to use music, and she spent a lot of 1 to 1 time with patients as well and we saw her … she kind of taught staff how to use music” (P1001, SM)  “[the music therapist] herself was just really good … in getting herself involved in the team.” (P20017, SM)  “we created like the … music care plans. So each patient's got it in their ward files, so it's at a glace we know what they like. It's there, it's easy access.” (P20035, SM)  “we've heard about music doing dementia patients really well, so yeah, it was actually nice to have somebody to actually put it in place... And give us the insight as to how it works, so to speak” (P20040, SM)   - *At site 1, only 4 SM attended a formal training session on the ward. This was changed to a voluntary workshop at site 2. The voluntary workshop was acceptable at site 2 with 10 SM attending across 2 workshops* - *90 SM interactions were reported across both sites by the MT. Most of these, 67%, were unplanned. Length of interactions varied with more, shorter interactions at site 1, and fewer, longer interactions at site 2. Time of interactions varied throughout the working day. From interventionist diaries, the MT attended the ward handover each day and then had multiple interactions with SM throughout the day.* - *22 FM interactions were reported across the two sites. At site 1 FM joined their relative in clinical sessions, with interactions increasing as the pilot progressed. Most interactions (75%) were unplanned* |
| **INCREASED COMMUNICATION AND UNDERSTANDING OF MUSIC TO SUPPORT PEOPLE WITH DEMENTIA (CMOC2, mechanism)** |
| “Seeing how beneficial that is, it makes us want to do it a bit more” (P20034, SM)  “We relate more to them as well” (P20042, SM)  “I think that when I start sharing more and more what I was doing during the session and the observation then sort of change a little bit… they started changing the reason for me to be with a patient.” (P20045, MT)  “So I do know this kind of activities really helped them, but when I have been here I have forgotten the previous things that I have done. So it was a refreshing thing that I was able to do them again.” (P1013, SM) |
| **STAFF AND FAMILIES USE TARGETED, PERSONALISED MUSIC TO MANAGE PATIENT DISTRESS AND REGULATE THE WARD ATMOSPHERE (CMOC2, outcome)** |
| “it unveils different characteristics and different behaviours of people that you probably wouldn't have been able to identify really” (P2005, SM)  “maybe a bit liberating, do you know. Because we've had instruments and we've kind of not really known how to use them” P20041, SM)  “So the music relaxes them, so it make it easier for us to give them personal care and look after them” (P20042, SM)  “I think music can be a way of connecting. So … it helps me relax and sort of feel more connected with them.” (P1003, SM)  “For me, feeling a sense of relief and seeing [my husband] having something that he can respond to now and again” (P1042, FM)   - *Trend in improved recognition of personhood (ADQ) for FM post-intervention, maintained at follow-up* - *Trend in improved somatic symptoms and anxiety/insomnia (GHQ) for FM post-intervention, maintained at follow-up* - *No changes in burnout (MBI), job satisfaction (IJS) or approaches to dementia (ADQ) shown for SM* - *Trend in reduced disruptiveness of neuropsychiatric symptoms (NPI) reported by staff post-intervention, not maintained at follow up* |
| **WHEN A MUSIC THERAPIST IS INTEGRATED INTO THE MDT (CMOC3, context)** |
| “Person who is leading the team, they should be on the board with the team.” (P1041, SM)  “I think the handovers, the care plans that have been created … And probably discussions as a team and involving family” (P2005, SM)  “I think by doing it for a long period of time so it starts to become a part of the culture … you would see a dramatic effect for patients and staff” (P1042, FM)  “I think even the staff that were a bit reluctant to pick up the tambourines and get involved, got involved by the end of it.” (P20017, SM)  “I think it helped that we already use music and we understand the importance of music.” (P20017, SM)   - *90 SM interactions were reported across both sites by the MT. Most of these, 67%, were unplanned. Length of interactions varied with more, shorter interactions at site 1, and fewer, longer interactions at site 2. Time of interactions varied throughout the working day. From interventionist diaries, the MT attended the ward handover each day and then had multiple interactions with SM throughout the day, most of which were unplanned* - *The MTs met with the ward managers regularly at both sites* - *Number of assigned MELODIC Champions was increased to a minimum of 2 at site 2 following feedback from site 1 that it was not always possible to meet with the Champion due to shift patterns. This was reported to be acceptable at site 2 with more regular communication* - *Musical care plans were created for all patients recruited into the study at both sites. Separate folders were created for the care plans. At site 2 copies were also placed in patient bedrooms, reported to support visibility* - *The MT was involved in ward handovers on intervention days, reporting increased interest from SM around ways to implement music and requests for support and assessments as the pilot progressed. The MT sent weekly reports to the ward manager and MELODIC Champion at both sites to be incorporated into the weekly MDT. Feedback to the MT following the meeting was lacking at both sites* |
| **Barriers (CMOC3, context)** |
| “you don't exactly know what's been going on with them all the time.” (P1026, FM)  “as a barrier I'd say the business and the acuity of the ward has shot up in the last week and a bit.” (P1007, SM)  “what I was receiving from staff was just that they were just like overwhelmed.” (P1043, MT)  “I don't think that they felt that it was part of their role as either clinician or health professionals to put in place it.” (P20045, MT)  “So they tried that [sedative medication], but not … try preventative intervention.” (P20045, MT)  “But then the next step to implement those actions into the daily life of patients is not made.” (P20045, MT)   - *91% of FM interactions occurred at site 1 where families were able to visit the ward openly. Only 2 FM interactions were reported at site 2 where FM had to book a time to visit their relative in a separate visiting room* - *Levels of staff absence and numbers of bank and agency staff were consistently high across the 3 months of data collection at both sites* |
| **MUSIC BECOMES PART OF ALL STAFF MEMBER’S ROLE (CMOC3, mechanism)** |
| “[Families] were very happy to provide feedback if it was needed and to share their knowledge about what that patient might like, what music they like and how to support them best.” (P1001, SM)  “I think it's someone coming in with shared values.” (ward manager, site 2)  “It's about everybody being involved and everybody being a team” (P20035, SM)  “I think those type of things from the music therapy can be adapted and like adopted into our other work as well. So it kind of all is a bit cohesive.” (P20017, SM) |
| **PERSONALISED MUSIC IS PART OF THE WARD CULTURE (CMOC3, outcome)** |
| “So I think it's one of the easier engagements we can do that you don't really have to think about and it, people respond really well to it” (P20035, SM)  “someone from the externals said that this is important, they feel more entitled to use it like they're not doing something silly” (P20045, MT)  “I do notice the effect of people coming into the space and like playing music, it's quite a strong effect.” (P1003, SM)   - *Ward managers and nurse consultants anecdotally reported an increase in SM citing music as an intervention they had tried when submitting incident reports post-intervention, maintained at follow-up. They also reported an increase in SM using music during 1:1 observations* - *Ward managers at both sites sought funding to continue employing an MT following the pilot* |


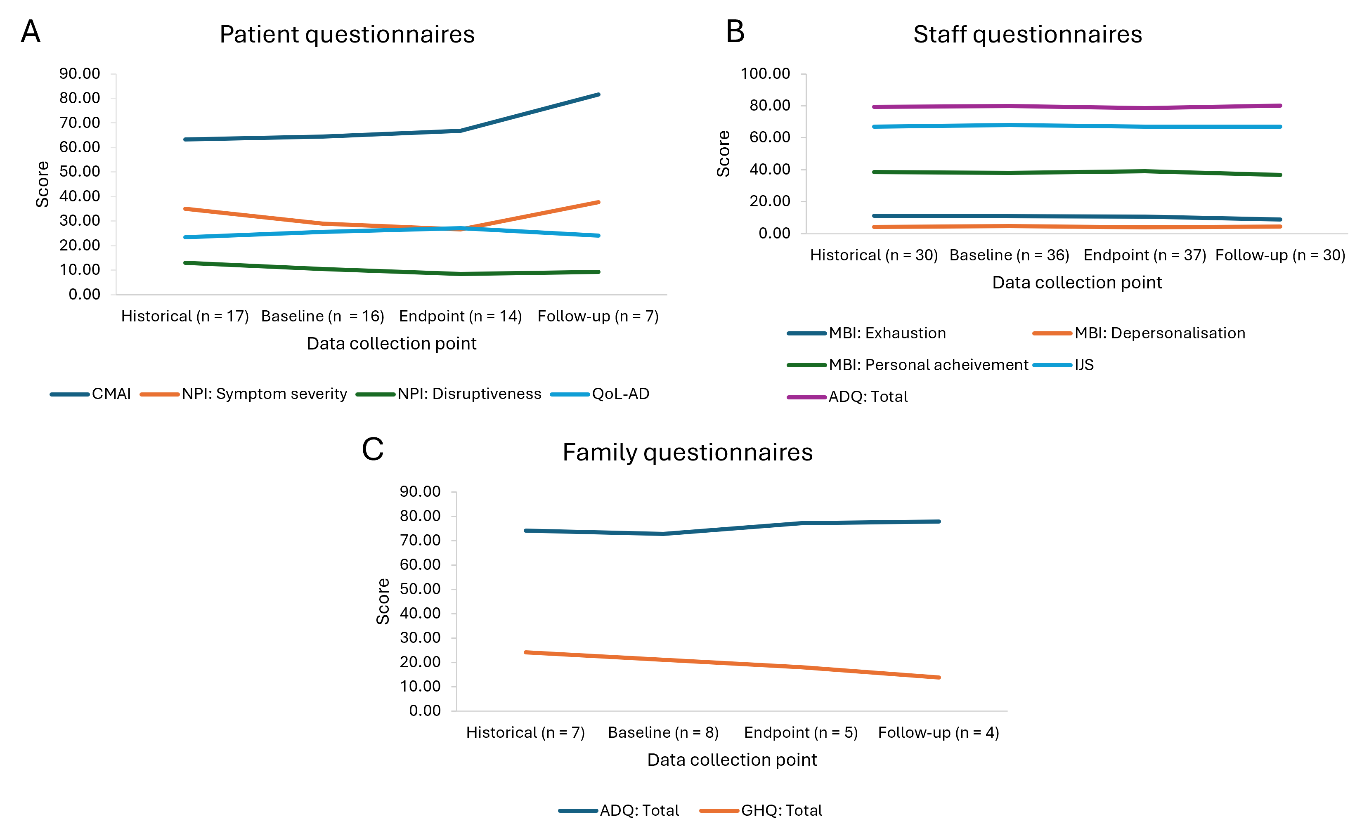


**Figure 1 Mean scores for patient (A), staff (B) and family (C) questionnaires across 4 data collection timepoints.** N numbers vary across timepoints as open cohort recruitment was used. CMAI = Cohen-Mansfield Agitation Inventory, NPI = Neuropsychiatric Inventory, QoL-AD = Quality of Life in Alzheimer’s Disease, ADQ = Approaches to Dementia Questionnaire, GHQ = General Health Questionnaire, IJS = Index of Job Satisfaction, MBI = Maslach Burnout Inventory.(1–7)
